# Job satisfaction of registered nurses in a private critical care unit in the Eastern Cape: A pilot study

**DOI:** 10.4102/hsag.v25i0.1345

**Published:** 2020-11-27

**Authors:** David Morton, Candice Bowers, Lauren Wessels, Angelique Koen, Juanita Tobias

**Affiliations:** 1 Department of Nursing Science, Faculty of Health Sciences, Nelson Mandela University, South Africa; 2 Private Practice, Port Elizabeth, South Africa

**Keywords:** job satisfaction, critical care, intensive care unit, professional nurses, private critical care unit

## Abstract

**Background:**

Job satisfaction is influenced by factors that are interpersonal (between nurse and colleagues), intrapersonal (within the nurse) and extra-personal (external to the nurse).

**Aim:**

The primary objective of this study was to explore and describe factors influencing the job satisfaction of registered nurses in a particular private critical care unit. The second objective was to make recommendations to enhance the job satisfaction of registered nurses in this private critical care unit.

**Setting:**

The population consisted of registered nurses in a private critical care unit in the Eastern Cape.

**Methods:**

This study utilised a quantitative descriptive design. Self-administered questionnaires were distributed amongst registered nurses in the critical care unit. Data were analysed and illustrated through tables.

**Results:**

Altogether, 39 registered nurses took part in the study. The majority of the participants (82%; *n* = 32) indicated that they enjoyed working with their team members. In addition, it was apparent that the majority (79%; *n* = 30) felt that they were sufficiently trained. Staff members felt that they had management support and felt satisfied at their workplace. Areas of concern included salaries, leave, debriefing and recognition.

**Conclusion:**

The majority of the registered nurses in this private critical care unit were extremely satisfied with their job. However, there were areas where this could be improved. The high levels of satisfaction at this single critical care unit lead to the question whether this situation is common throughout the Eastern Cape, which opens the path for further research in this regard.

## Background

Critically ill patients are cared for in intensive care units (ICUs) that are staffed by specialist personnel who are equipped with a range of life-support technologies. In other words, specialised procedures and equipment are managed by skilled healthcare practitioners who can heal and sustain life that would otherwise be threatened (Matlakala & Botha 2016:50). Frequently, ICU nurses have to make life-saving decisions requiring sound theoretical knowledge and critical thinking skills (Morton & Fontaine 2018:2). However, the conditions in which critical care nurses work can be best described as adverse, which plays a role in undermining their job satisfaction.

Currently, South Africa has 8.9 ICU beds per 100 000 population unlike the United States of America that has 30 ICU beds per 100 000 population (Anesi et al. 2018:2). The most recent available statistics indicate that in 2012 South Africa had 4168 critical care and high care beds managed by 4584 professional nurses (Pretorius & Klopper 2012:67). Joynt et al. (2019:617) pointed out that of this total of 4168 ICU and high care beds, 57% were in the private sector. Furthermore, they noted that there are significant resource shortages in South Africa, especially in the form of ICU bed numbers and trained nurses or intensivists (Joynt et al. 2019:618).

However, despite the great need for trained critical care nurses, Adams, Chamberlain and Giles (2018) cited a number of sources, indicating that hospital managers around the world are finding the retention and recruitment of skilled critical care nurses to be a serious challenge. This is partly because of the highly specialised skills required by such nurses, as well as the demanding working environment (Hauck et al. 2011; Pretorius & Klopper 2012:67). These working conditions, which are often adverse, have a direct impact on the job satisfaction of critical care nurses.

Institutions place a great emphasis on the job satisfaction of their employees as they spend a great deal of their time in the workplace. Baum and Kagan (2015:213) argued that job satisfaction amongst nurses has an effect on whether or not they remain in the profession or whether they remain in the healthcare facility where they work. Job satisfaction is described as ‘how people feel about their jobs and different aspects of their jobs’ (Spector 1997:2). The concept of job satisfaction is strongly associated with the expectations of employees about their job and what they hope to gain from it (Hong Lu et al. 2012). According to Castaneda and Scanlan (2014:130), job satisfaction is a complex phenomenon that influences productivity, performance, absenteeism, retention, recruitment, organisational commitment, patient satisfaction and patient care.

According to a systematic review by Dilig-Ruiz et al. (2018:132), the job satisfaction of critical care nurses is influenced by employment factors, such as rotating shifts, job stress and burnout (emotional exhaustion). In addition, it is determined by organisational factors, such as personnel resources, staffing, teamwork and cohesion. Manyisa and Van Aswegen (2017) indicated that the working conditions of nurses are affected by shortages of human and material resources as well as poor physical infrastructure. Indeed, amongst other factors, poor hospital infrastructure was found to be a major contributor to South African health professionals exploring the possibility of emigration (Labonté et al. 2015).

Job satisfaction is a complex concept that is influenced by extrinsic and intrinsic factors. Herzberg (1968) in his two-factor theory identifies hygiene factors (extrinsic factors) and motivational factors (intrinsic factors) that influence an individual’s job satisfaction. Extrinsic factors for professional nurses include the tangible aspects of work, such as salary, benefits and bonuses, whilst intrinsic factors include personal and professional development opportunities, status and recognition (Klopper et al. 2012:693). Atefi et al. (2014:355) emphasised that personal values and beliefs, work environmental factors and motivational factors are associated with the job satisfaction of critical care nurses.

Labonté et al. (2015:13) claimed that in relation to other healthcare practitioners, South African nurses are most likely to report plans to emigrate in the short term, are the least supportive of community service and are the least satisfied with their salaries. Additional factors that decrease job satisfaction amongst nurses in South Africa are increased patients loads, human immunodeficiency virus (HIV) and acquired immune deficiency syndrome (AIDS) epidemic, long working hours, shift work, physical infrastructure and staff shortages (Manyisa & Van Aswegen 2017:37). Hence, Lu, Zhao and While (2019:30) argued that it is essential to increase nurses’ job satisfaction as this can potentially improve patients’ perceptions of the quality care they receive and also improve the chances of increasing the nursing workforce.

## Problem statement

After informal discussions with registered nurses in the private critical care unit where a number of researchers were working, certain issues became apparent. There was a shortage of trained critical care nurses in the unit, which is supported by research conducted by Joynt et al. (2019:618). In addition, there was an increase in workload, which was challenging for the staff members regarding productivity (Matlakala, Bezuidenhout & Botha 2014:4). Some staff members were also given opportunities to further their studies, which, in turn, placed strain on the remaining staff in the unit. In addition, it was observed that there had been many modifications in the unit through the adoption of evidence-based practices, which appeared to increase the stress levels of staff. There are few recent studies that focused specifically on the job satisfaction of ICU nurses in the private sector in South Africa and none in the Eastern Cape that the researchers are aware of.

## Research questions

This study sought to find answers to the following questions:

- What is the job satisfaction of registered nurses in a private critical care unit?
- What recommendations can be made to enhance the job satisfaction of registered nurses in the private critical care unit under study?

## Definition of concepts

A *critical care unit* is an environment whereby patients with actual or potential life-threatening illness or injury are managed and cared for. These patients require vigilant monitoring (Urden, Stacey & Lough 2010:2). The study described in this article was conducted in a critical care unit in a private hospital.

*Job satisfaction* can be considered as a global feeling about a job or as a related constellation of attitudes about various aspects or facets of a job (Hong Lu et al. 2012). The study investigated the job satisfaction of registered nurses working in a private critical care unit.

A *registered nurse*, according to the *Nursing Act no. 33* of 2005 (South Africa 2005), is a person who is registered and licensed as a registered nurse under this *Act*. A registered nurse is an educated, competent and independent nursing practitioner who assumes responsibility and accountability for such practice. The study sought to describe the job satisfaction of registered nurses working in a private critical care unit.

A *private hospital*, according to the *Ministry of Legal Affairs Act 22(4)*, refers to an institution where persons suffering from any sickness, injury or infirmity are given medical treatment and nursing care that is operated by a privately owned company. Medical aid and cash are exchanged for services rendered. The study was conducted in a critical care unit in a private hospital.

## Theoretical framework

Herzberg’s (1968) two-factor theory of career and job satisfaction was used as the theoretical framework for this study. The theory concerns a person’s two-dimensional need system. It proposes that an individual is influenced by both motivational (intrinsic) and hygiene (extrinsic) factors (Table 1). Motivational factors refer to elements that generate satisfaction from within the individual, such as being innovative and creative in his or her job. Motivational factors (intrinsic), as the term implies, are generally involved in inspiring employees. Examples of these determiners of job satisfaction are promotion, provision of more responsibility, being responsible for one’s own practice, having the opportunity to do one’s job creatively and innovatively, recognition, and achievements (see Table 1). On the contrary, hygiene (extrinsic) factors refer to elements that generate job satisfaction from outside the individual, such as nursing supervision, hospital policy and administration, working conditions and interpersonal relations between nurse colleagues (Lephalala, Ehlers & Oosthuizen 2008:61).

**TABLE 1:** Intrinsic and extrinsic factors.

Intrinsic factors influencing job satisfaction	Extrinsic factors influencing job satisfaction
Achievements	Working conditions
Recognition	Salary
Responsibility	Organisation or administration policies
The nature of work itself	Supervision
Advancement (promotions)	Interpersonal relations

*Source:* Lephalala, R.P., Ehlers, V.J. & Oosthuizen, M.J., 2008, ‘Factors influencing nurses’ job satisfaction in selected private hospitals in England’, *Curationis* 31(3), 60–69.

## Research design and method

In this study, a quantitative, descriptive research design was followed in the exploration of the job satisfaction of registered nurses in a mixed medical–surgical private critical care unit in Port Elizabeth, Eastern Cape, South Africa. The critical care unit comprised a 23-bedded unit: eight of these beds were cardio thoracic, four were high care beds, four were isolation beds and then a further seven beds.

### Population and sampling

According to Brink, Van Der Walt and Van Rensburg (2012:131), a population is a group of people or objects that are of interest to the researcher. Thus, only those who met the criteria set by the researchers were studied. The research population for this study comprised 40 registered nurses who worked in a private critical care unit. The participants included registered nurses on duty and excluded those on annual leave, sick leave and study leave. Moreover, the registered nurses included permanently employed nurses and those working through agencies. The researchers utilised an all-inclusive sampling method for the study. Hence, all the registered nurses working in the private critical care unit who met the inclusion criteria were asked to participate in the study.

### Data collection

The data collection instrument for the study was in the form of a structured questionnaire. Two sources were found to be useful in the construction of the questionnaire in light of the research topic. The first was the study conducted by Ellenbecker and Byleckie (2005:70), who investigated home healthcare nurses’ job satisfaction. The second source that was used was a national campaign launched in 2006 called Advancing Excellence in America’s Nursing Homes (Advancing Excellence 2012). The questionnaire was then contextualised to the critical care unit and divided into three sections, namely, demographic data, extrinsic factors influencing job satisfaction and intrinsic factors influencing job satisfaction. The questionnaire consisted of a variety of questions, namely, open- or close-ended and Likert-scale questions.

Potential participants were approached individually by the researchers for the purposes of obtaining consent. The purpose of the research study and the process of completing the questionnaires were explained to the participants in the hope of reducing the chances of possible errors. When the participants agreed to participate in the study, they were requested to sign a consent form and supplied with a questionnaire. To allow participants to choose whether or not to participate, they were requested to place the completed anonymous questionnaire in a sealed collection box, which was left in the unit, to which only the researchers had access. The questionnaires were collected 7 days later to accommodate all staff on the dayshift and nightshift. Of the 40 questionnaires handed out, 39 were returned, yielding a response rate of 97.5%.

### Data analysis

The data obtained from the questionnaire were analysed using Microsoft Excel. Descriptive statistical analyses were conducted and frequency distributions were used to analyse the data.

### Reliability and validity

To enhance the reliability and validity of the study, the researchers conducted a literature review as part of the process of developing the data collection instrument. The questionnaire was tested on two nurses to help determine that it measured what it was intended to measure, which enhanced the reliability of the questionnaire. The researchers, as experienced critical care nurses, were able to assess the questionnaire for face validity and content validity.

### Ethical considerations

Ethical approval to conduct the research was obtained from an authorised higher education institution. Access to the hospital was sought through the private hospital’s chief executive’s office with the unit managers acting as gatekeepers. The potential participants were informed regarding the title, purpose, objectives and methods of the study to facilitate the request to participate. Care was also taken to explain their rights to voluntary participation, privacy and confidentiality.

## Results

The socio-demographic characteristics of the participants are presented in Table 2, whilst the analysis of the extrinsic and intrinsic job satisfaction factors is given in Tables 3 and 4, respectively. Moreover, the data are presented under the following headings: socio-demographic characteristics, extrinsic job satisfaction factors and intrinsic factors job satisfaction factors.

**TABLE 2:** Socio-demographic characteristics of the participants.

Demographic characteristics	Total: *n* = 39	Total: % = 100
**How many years have you been working as a registered nurse in the critical care unit?**		
< 2 years	9	23
2–5 years	7	18
6–10 years	12	31
11–15 years	1	2
16+ years	10	26
**Are you working as one of the following?**		
Permanent employed registered nurse	27	69
Clinical facilitator or mentor	0	0
Shift leader	4	10
Agency worker	8	21
**Do you hold an additional qualification registered with SANC?**		
Yes	18	46
No	21	54
**Critical care or ICU-trained qualified**	14	36

*Source:* Lephalala, R.P., Ehlers, V.J. & Oosthuizen, M.J., 2008, ‘Factors influencing nurses’ job satisfaction in selected private hospitals in England’, *Curationis* 31(3), 60–69.

SANC, South African Nursing Council; ICU, intensive care unit.

**TABLE 3:** Extrinsic (hygiene) factors influencing nurses’ levels of no job satisfaction.

Hygiene (extrinsic) factors	Disagree		Agree	
	*n*	%	*n*	%
**1. Working conditions**				
Physical working conditions (heat, light, dust, noise, cleanliness, etc.) are generally satisfactory.	5	13	24	62
**2. Salary**				
I am satisfied with my current level of pay and benefits.	11	29	14	37
A workplace incentive programme is available.	18	47	8	21
**3. Organisation or administration policies**				
The company’s leave policy is practical.	9	23	18	48
My employer provides team-building programmes.	12	31	11	29
I have received the training I need to perform my duties competently and safely.	3	8	30	79
On-the-spot training is available.	5	13	28	74
Debriefing sessions are provided on a regular basis.	20	52	13	33
**4. Supervision**				
The managers I work for back me up.	5	13	26	71
Complaints and problems are handled fairly here.	5	13	24	65
There is a good spirit of cooperation between employees and management.	6	15	25	66
Management is hard to please.	11	30	16	43
Management believes that the well-being of their employees is important.	5	13	25	68
**5. Interpersonal relations**				
I enjoy working with team members in my unit.	0	0	32	82
There is a friendly atmosphere at work.	4	10	29	77
There is a good spirit of cooperation between employees and management.	6	15	25	66

*Source:* Adapted from Lephalala, R.P., Ehlers, V.J. & Oosthuizen, M.J., 2008, ‘Factors influencing nurses’ job satisfaction in selected private hospitals in England’, *Curationis* 31(3), 60–69.

**TABLE 4:** Intrinsic (motivating) factors influencing nurses’ job satisfaction.

Motivating (intrinsic) factors	Disagree		Agree	
	*n*	%	*n*	%
**1. Achievements**				
After a day’s work, I feel like I accomplished something.	4	10	33	85
My duties at work are stimulating.	3	8	32	89
Most days I find my job to be extremely satisfying.	4	10	19	72
**2. Recognition**				
Physicians value my input.	7	18	21	55
Acknowledgement for individual achievements is provided.	11	29	18	47
I feel valued by my employer and organisation.	4	10	26	67
**3. Responsibility**				
I am involved in patient care decision-making processes.	2	5	33	87
My opinions are considered when changes are made at work.	8	21	21	55
**4. Nature of the job**				
My job description accurately reflects the duties that I perform.	3	8	33	87
I feel overwhelmed by the job.	20	53	9	23
I feel stressed at my workplace.	20	51	5	13
I hate to get up in the morning to go to work.	27	69	2	5
**5. Advancement**				
I feel as if I am in a ‘dead end’ job.	31	79	3	8
I am provided opportunities for growth and development.	2	6	27	75

*Source:* Lephalala, R.P., Ehlers, V.J. & Oosthuizen, M.J., 2008, ‘Factors influencing nurses’ job satisfaction in selected private hospitals in England’, *Curationis* 31(3), 60–69.

### Socio-demographic characteristics

A total of 31% (*n* = 12) of the participants had worked in the critical care unit for 6–10 years, and 26% (*n* = 10) had more than 16 years’ experience. Hence, more than half of the participants had over 6 years of experience in the critical care unit.

The majority of staff members in the critical care unit were permanently employed. Hence, of all the participants (69%; *n* = 27) were permanently employed in the critical care unit. A smaller percentage of participants (21%; *n* = 8) were agency staff. Regarding the training of the participants, 54% (*n* = 21) did not have an additional qualification with the South African Nursing Council (SANC). Furthermore, only one-third of the participants (36%; *n* = 14) were trained in critical care.

### Hygiene or extrinsic factors and job satisfaction

Herzberg (1973) uses the term hygiene (or extrinsic factors) to describe the job factors that are considered to be maintenance factors that help prevent dissatisfaction with work; however, these do not necessarily motivate or provide satisfaction (Purohit & Bandyopadhyay 2014:3). The hygiene factors as they relate to job satisfaction are shown in Table 3.

#### Working conditions

The majority of the participants (62%; *n* = 24) agreed that the physical working conditions, such as heat, light, dust, noise and cleanliness, were generally satisfactory. Altogether 24% (*n* = 9) of the participants remained neutral concerning this statement.

#### Salary

The participants were asked about their satisfaction with their current level of pay and benefits. Just over one-third (37%; *n* = 14) of the participants indicated that they were satisfied with their current level of pay and benefits. However, 29% (*n* = 11) of the nurses indicated that they were not, and a further 34% (*n* = 13) gave a neutral response. Concerning the availability of a workplace incentive programme, 47% (*n* = 18) disagreed that there was one available, whilst 32% (*n* = 12) expressed neutrality and 21% (*n* = 8) agreed with the statement.

#### Organisation or administration policies

Concerning the practicality of the company’s leave policy, nearly a quarter of the participants (23%; *n* = 9) disagreed with the practicality of the leave policy. However, 40% (*n* = 15) agreed with this statement, with over a quarter of the participants (29%; *n* = 11) remaining neutral. A small proportion of participants (28%; *n* = 11) agreed that the employer provided team-building programmes for their staff members. Over one-third (38%; *n* = 15) of participants indicated neutrality regarding the statement, whilst 26% (*n* = 10) disagreed that the employer provided team-building programmes. The majority of the participants (79%; *n* = 30) stated that they had received the training they needed to perform their duties competently and safely. Only a small percentage (8%; *n* = 3) of participants stated that they had not. In addition, 74% (*n* = 28) of the participants mentioned that on-the-spot training was available to them, with only 13% (*n* = 5) disagreeing with the statement. Of the participants, 52% (*n* = 20) strongly disagreed or disagreed that debriefing sessions were provided on a regular basis. Hence, only 33% (*n* = 13) of the participants agreed that debriefing sessions were provided on a regular basis.

#### Supervision (hygiene)

Related to this statement was the importance that employees placed on knowing that their managers would back them up in a difficult situation. A total of 41% (*n* = 15) of participants agreed and 30% (*n* = 11) strongly agreed that their managers would back them up. Also linked to this point was whether complaints and problems were handled fairly. The majority of the participants agreed (41%, *n* = 15) or strongly agreed (24%, *n* = 9) with this statement. An important extrinsic factor concerned cooperation between employees and management. Nearly two-thirds of the participants felt that there was a good spirit of cooperation between employees and management in the critical care unit. However, regarding whether management was hard to please, 43% (*n* = 16) agreed or strongly agreed that management was hard to please. However, 30% of the participants (*n* = 11) disagreed with this statement. Regarding the statement whether or not management believed that the well-being of their employees was important, a majority of 54% (*n* = 20) of the participants agreed. However, 19% (*n* = 7) felt neutral about the statement and 13% (*n* = 5) disagreed with this statement.

#### Interpersonal relations (hygiene)

The majority of the participants (82%; *n* = 32) agreed or strongly agreed that they enjoyed working with the team members in their unit. Only 18% (*n* = 7) of the participants remained neutral regarding this statement. In relation to experiencing a friendly atmosphere at work, 40% (*n* = 15) of the participants strongly agreed and 37% (*n* = 14) agreed to the statement. However, almost a quarter (*n* = 10) of the participants disagreed with this statement. Of the participants, 21% (*n* = 8) strongly agreed and 45% (*n* = 17) agreed that there was a good spirit of cooperation.

### Influence of intrinsic or motivating factors on job satisfaction

Herzberg (1968) used the term ‘motivating’ (or intrinsic factors) to describe the job factors that are considered as bringing about satisfaction with work, and thus leading to, promoting or producing satisfaction (Purohit & Bandyopadhyay 2014:3). These factors are discussed under the following categories: (1) Achievements, (2) Recognition, (3) Responsibility, (4) Nature of the job and (5) Advancement. The results concerning the intrinsic factors that influenced the job satisfaction of the participants are presented in Table 4.

#### Achievements

Regarding the statement whether after a day’s work, they felt that they have accomplished something, 85% (*n* = 33) of the participants agreed or strongly agreed. Furthermore, a large majority of 89% (*n* = 32) of the participants felt that their duties were stimulating. When asked about whether their job was extremely satisfying most days, a majority of 72% (*n* = 19) of the participants agreed or strongly agreed with this statement. Only 18% (*n* = 7) indicated that they felt neutral regarding the statement, and a mere 10% (*n* = 4) disagreed or strongly disagreed with the statement.

#### Recognition

The majority of the participants felt that physicians valued their input, with 55% (*n* = 21) of the participants agreeing or strongly agreeing with this statement. Only 26% (*n* = 10) stated that they felt neutral about physicians valuing their input, whilst 18% (*n* = 7) disagreed or strongly disagreed. Regarding acknowledgement for individual achievements, 47% (*n* = 18) of participants felt that they were acknowledged, whilst 29% (*n* = 11) felt neutral regarding feeling acknowledged for individual achievements. The majority of the participants felt valued by their employer and organisation, with 67% (*n* = 26) responding in the affirmative. Some participants (23%; *n* = 9) remained neutral with regard to this statement, whilst a minority (10%; *n* = 4) disagreed or strongly disagreed.

#### Responsibility

The participants were asked about their involvement in the patient care decision-making processes. The majority of the participants (87%; *n* = 33) agreed or strongly agreed that they were indeed involved in patient care decision-making. Regarding the statement whether staff members’ opinions were considered when changes were made at work, 55% (*n* = 21) agreed or strongly agreed with this statement. Hence, the majority of the participants agreed that their opinions were considered when changes were made at work.

#### Nature of the job

A majority of 87% (*n* = 33) of the participants agreed that their job description accurately reflected the duties that they performed. Only 8% (*n* = 3) disagreed with this statement. A related question concerned whether they felt overwhelmed with their job. A total of 53% (*n* = 20) of the participants disagreed or strongly disagreed, whilst 24% (*n* = 9) indicated that they felt neutral regarding the statement. However, 23% (*n* = 9) of the participants agreed or strongly agreed that they felt overwhelmed by their job. In addition, a high percentage of (51%; *n* = 20) of the participants disagreed that they felt stressed in their workplace. Only 13% (*n* = 5) of the participants agreed that they felt stressed at their work place. However, one-third of the participants (33%; *n* = 13) took a neutral stance regarding this question. Only 5% (*n* = 2) of the participants agreed with the statement ‘I hate to get up in the morning to go to work’. However, 69% (*n* = 27) of participants disagreed or strongly disagreed that they hated to get up in the morning to go to work. Notably, over a quarter of the participants (26%; *n* = 10) indicated a neutral response regarding this statement.

#### Advancement

With regard to the factor of advancement, the results showed that 79% (*n* = 31) of the participants disagreed with the statement that they were in a dead end job, whilst 13% (*n* = 5) of the participants indicated that they felt neutral regarding the statement. Only 8% (*n* = 3) of the participants strongly agreed that they felt as if they were in a dead end job. A majority of 75% (*n* = 27) of the participants felt that they were provided with opportunities for job growth and development. Only a small percentage of participants (6%; *n* = 2) disagreed with this statement.

## Discussion

The socio-demographic characteristics of the participants revealed that almost 60% of the critical care nurses had 6 years or more experience in the critical care unit. Hence, the majority of the participants had substantial experience of having worked in the critical care unit, which indicated that the staff turnover for the critical care unit was low, as staff members tended to remain in the unit for an extended period of time. Hong Lu et al. (2012:101) cited a number of sources that support the argument that a low staff turnover is associated with job satisfaction. Thus, their staying for a significant length of time in their jobs indicates that there was a sense of job satisfaction amongst many of the critical care nurses in the private critical care unit under study. In fact, over two-thirds of the participants stated that they found their job ‘extremely satisfying’. Most of the critical care staff members were permanently employed, with a small minority consisting of agency workers.

### Extrinsic factors influencing job satisfaction

The majority of study participants felt that the physical working conditions of the critical care unit in which they worked were generally satisfactory. However, over one-third either remained neutral or disagreed that the physical working conditions were satisfactory. Manyisa and Van Aswegen (2017) highlighted that working conditions had a significant impact on the job satisfaction of nurses.

Pay and benefits were a contentious issue amongst the critical care nurses under study, with one-third of participants being satisfied with their level of pay and benefits. However, one-third of participants were not satisfied, and more than one-third took a neutral stance regarding pay and benefits. Hong Lu et al. (2012:102) stated that globally nurses are largely dissatisfied with remuneration and issues around job promotion. Critical care nurses in Klopper et al.’s (2012:690) South African study also indicated dissatisfaction with their salaries. Similarly, most of the participants in this study disagreed or were unsure of the availability of a workplace incentive programme in the critical care unit.

Most of the participants were either unsure or in disagreement with the leave policy. The issue of leave was a factor that was associated with job dissatisfaction in Klopper et al.’s (2012:690) study. However, this dissatisfaction with leave did not prevent the majority of participants from indicating that they were extremely satisfied with their work most days. Most of the participants were not satisfied with the organisation’s provision of team-building programmes, which is problematic as such programmes build team commitment and is strongly associated with job satisfaction (Galletta et al. 2016). Another negative factor was that most of the participants indicated that debriefing did not occur in the unit. Discussing debriefing sessions, Flannery, Ramjan and Peters (2016:97) pointed out that nurses are sometimes required to make decisions for patients who may be unable to participate in the decision-making process. This can be emotionally challenging and, ideally, debriefing sessions should be provided thereafter. Despite the issue of leave, the lack of team-building programmes and the lack of debriefing in the unit, the majority of participants stated that they were extremely satisfied with their work.

Regarding training, most of the participants indicated that they had received the necessary training to perform their duties competently and safely. In addition, the majority of the participants stated that on-the-spot training was available to them. Sojane, Klopper and Coetzee (2016:9) argued that the training of nurses is necessary to provide them strategies and coping skills to enhance their practice environment that will have a positive affect on their job satisfaction. Hence, this might explain why the majority of the participants were satisfied with their work.

The majority of the participants indicated that their managers would back them up in a difficult situation. In addition, a large number of participants agreed that complaints and problems were handled fairly in the unit they worked. Nearly two-thirds of the participants felt that there was a good spirit of cooperation between employees and management in the critical care unit. Furthermore, over half of the participants indicated that management felt that the well-being of their employees was important. However, less than half of the participants felt that management was hard to please. Moreover, almost one-third of the participants disagreed with this statement. Sellgren, Ekvall and Tomson (2008:585) emphasised that the leadership behaviour of nurse managers is an important variable influencing the job satisfaction of nurses. Based on the participants’ responses, it appears that the nurse managers of this critical care unit were providing effective leadership and management.

The majority of the participants agreed that they enjoyed working with the team members in their unit. Related to this was that the majority of the participants indicated that there was a friendly atmosphere at work. In addition, the majority of the participants agreed that there was a good spirit of cooperation between employees and management. Castaneda and Scanlan (2014:133) stated that relationships with other members of the nursing staff, collaboration with medical staff and communication between nurses and physicians are important factors in creating job satisfaction.

### Intrinsic factors influencing job satisfaction

A majority of the participants felt that their job was extremely satisfying most days. As such, most of the participants felt that they had accomplished something after a day’s work and that they felt that their duties were stimulating. Atefi et al. (2014:11) maintained that nurses feel honoured and happy when they help patients. Giving holistic care and, in particular, being able to identify the various needs of their patients is another important factor in nurses’ job satisfaction. In the study described in this article, only a small number of participants expressed feeling stressed at their workplace.

Despite a majority of the participants agreeing that the physicians valued their input, quite a number of participants did not feel that this was the case or they were undecided. However, a high percentage of participants felt that they were involved in patient care decision-making processes and that their opinions were considered when changes were made at work. Mokoka, Oosthuizen and Ehlers (2010:107) argued that nurses want to be appreciated and respected by management and doctors. They also want their expertise to be recognised, and want to participate in decision-making processes pertaining to patient care. A large number of participants felt that they were not acknowledged for individual achievements, although a higher percentage felt that they were acknowledged. Recognition from managers plays an important role with regard to nurses’ job satisfaction (Hong Lu et al. 2012:1020). Sojane et al. (2016:9) recommended in-service training for nursing managers to equip them with the skills to express recognition and praise for those staff under their supervision.

A majority of participants indicated that they did not feel stressed at work. Lim, Bogossian and Ahern (2010:23) identified a high workload as the main stressor for critical care nurses. However, a small percentage of participants indicated that they hated getting up in the morning to go to work, whereas a large percentage of participants disagreed with this statement, indicating a positive attitude towards their vocation. According to Castaneda and Scanlan (2014:132), personal factors, such as an individual’s needs and aspirations, determine this attitude, as well as group and organisational factors, such as working conditions, compensation, and relationships with co-workers and supervisors.

The findings of this study were consistent with those of Breau and Rheaume (2014:23), as a high percentage of participants stated that they were provided with opportunities for growth and development. However, Joynt et al. (2019) stated that, in South Africa, only 25.6% of nurses are ICU-trained. In addition, most of the participants said that they received ‘on-the-spot’ training. Sabanciogullari and Dogan (2014:854) argued that nurses who do not sufficiently develop professional identities during their vocational education can still develop them in the workplace. They maintain that interventions aimed at increasing job satisfaction and the will to keep up with the job contribute to the nurses developing their vocational skills. This appears to relate to the response of most of the participants who indicated that they felt extremely satisfied with their job.

## Limitations of the study

As this was a pilot study, it focused only on one private critical care unit in the Eastern Cape. The results were not intended to be generalised to other health facilities. It was found that some questions on the questionnaire may not have been applicable to certain agency staff members. A further limitation, from an ethical perspective, was that because individuals were personally asked if they would take part in the study, they may have felt obliged to do so. However, as participants were requested to insert the questionnaire in a sealed box anonymously, it allowed them the choice whether or not to take part and the researchers were not able to determine who completed a questionnaire.

## Recommendations

It is recommended that nurse managers include critical care nurses in decision-making pertaining to nursing practice or patient care. Furthermore, a recognition or reward programme could help make staff feel that they are appreciated. Staff education sessions on stress-relieving techniques and time management would be beneficial to critical care staff. An increase in annual leave days was highlighted by the participants and this may have a positive affect on the quality of care. The provision of debriefing sessions in the critical care unit would benefit the staff members. Also encouraging the utilisation and awareness of the employee wellness programme, which is available to staff members at the institution, would be beneficial to enhance job satisfaction.

Concerning future research, this pilot study has highlighted the need for a study on a larger scale to be conducted at private critical care units across the Eastern Cape. However, it may be of benefit to the study to include a qualitative component to complement the quantitative findings as certain more nuanced aspects of job satisfaction of ICU nurses cannot be elicited from a quantitative study alone. Considering that agency nurses play a significant role in critical care, it may be of benefit that a separate study is conducted on a population of agency staff with a questionnaire tailored to their working situation.

## Conclusion

The findings of this study show that the majority of the participants were extremely satisfied with their job in the private critical care unit. However, it was clear from the findings that this study highlighted some interesting job-related factors that require further exploration in relation to job satisfaction. These include extrinsic factors such as the need for improved salaries and incentives, as well as the need for training and debriefing. An important intrinsic factor is the need for critical care nurses to receive recognition. The participants of this study experienced high levels of job satisfaction in a single critical care unit. As such, this study will be enhanced if it is undertaken on a larger scale to explore whether the findings are reflective of a broader trend across the private healthcare group or if it reflects the job satisfaction of registered nurses in this unit alone.
